# Intrinsically active MEK variants are differentially regulated by proteinases and phosphatases

**DOI:** 10.1038/s41598-018-30202-5

**Published:** 2018-08-07

**Authors:** Merav Ordan, Chiara Pallara, Galia Maik-Rachline, Tamar Hanoch, Francesco Luigi Gervasio, Fabian Glaser, Juan Fernandez-Recio, Rony Seger

**Affiliations:** 1 Department of Biological Regulation, Weizmann Institute of Science, Rehovot, Israel; 2 Life Sciences Department, Barcelona Supercomputing Center, Barcelona, Spain; 3 Department of Chemistry, University College London, London, UK; 4 Bioinformatics Knowledge Unit, Technion, Haifa, Israel; 5 Institut de Biologia Molecular de Barcelona, CSIC, Barcelona, Spain

## Abstract

MAPK/ERK kinase (MEK) 1/2 are central signaling proteins that serve as specificity determinants of the MAPK/ERK cascade. More than twenty activating mutations have been reported for MEK1/2, and many of them are known to cause diseases such as cancers, arteriovenous malformation and RASopathies. Changes in their intrinsic activity do not seem to correlate with the severity of the diseases. Here we studied four MEK1/2 mutations using biochemical and molecular dynamic methods. Although the studied mutants elevated the activating phosphorylation of MEK they had no effect on the stimulated ERK1/2 phosphorylation. Studying the regulatory mechanism that may explain this lack of effect, we found that one type of mutation affects MEK stability and two types of mutations demonstrate a reduced sensitivity to PP2A. Together, our results indicate that some MEK mutations exert their function not only by their elevated intrinsic activity, but also by modulation of regulatory elements such as protein stability or dephosphorylation.

## Introduction

The Extracellular Signal-Regulated kinase 1/2 (ERK) cascade is one of the main signal transduction pathways conveying information from cell surface receptors into the cell (Reviewed in^1–3^). Signals initiated by these receptors are usually transmitted to ERK via a sequential activation of Ras, the protein kinases B/C-Raf (Raf) and MEK1/2 (MEK). ERK then phosphorylates a large number of substrates leading to the induction and regulation of many cellular processes, principal among which are proliferation and differentiation^4^. Being such a central signaling pathway, its dysregulation leads to various pathologies, mainly cancer. Indeed, activating mutations of Ras, Raf, MEK and even ERK serve as major oncogenes in more than 40% of all cancers, and ERK activation was reported in more than 85% of cancers^2,5,6^. In addition, MEK and Raf inhibitors have been in use as cancer treatment since 2010, mainly in mutated B-Raf melanoma^7^. Aside of cancer, the dysregulated cascade can participate in developmental diseases such as RASopathies (mainly Noonan, Costello and Cardio-Facio-Cutaneous syndrome (CFC)^8,9^), or Extracranial Arteriovenous Malformation (AVM^10^). Finally, elevated ERK activity was also implicated in the induction of neurological disorders^11^, diabetes^12^ and other diseases^2^.

MEK is one of the regulatory components in the ERK cascade^2^. It is a dual specificity protein kinase, able to phosphorylate both activatory Thr and Tyr residues in ERK^13^. Its phosphorylation targets repertoire is very limited, and almost all its activity is turned towards ERK^13^, although MyoD, β-arrestin 2, HSF1 and possibly also Raf were reported to serve as MEK substrates as well^14–17^. Interestingly, MEK affects other targets in a non-catalytic manner, including its association-dependent nuclear export of ERK^18^ and PPARγ^19^, formation of a transcriptional complex that represses MyoD transactivation^20^ and interacting with AKT to regulate FOXO1 localization^21^. Much information has been accumulated on the structure-function relationship of MEK. As would be expected, it contains all functionally critical regions shared within protein kinases, including an activation loop that keeps MEK inactive without stimulation^22^. MEK activation is achieved once its two activatory Ser residues within the activation loop (S218 and S222) are phosphorylated by an activating kinase. This activation can be mimicked by phosphomimetic mutations of the same two residues^23–25^. The phosphorylation opens the catalytic pocket, and keeps MEK active until it is dephosphorylated by PP2A^26^. This process resembles the activation of ERK, which is activated by MEK phosphorylation of its activatory residues, which are dephosphorylated by either PP2A and PTP-SL or dual specificity phosphatases^27,28^.

Although MEK’s structure is similar to that of many other protein kinases^22,23,29^, its basal activity is much lower compared to the other protein kinases. This is achieved by an N-lobe located α-helix (αA-helix), which has been reported to play a negative regulatory role on MEK1 catalytic activity^30^ related to the stabilization of αC-helix outward displacement^22^. This negative regulatory region maintains the low basal activity of MEK because it wraps around the protein, interacting with residues in the exterior of the kinase domain, and keeping the catalytic pocket firmly closed. Thus, activity of MEK can be the result not only of specific alteration of the activation loop, but also of deletions or mutations in the regulatory αA-helix or the kinase domain residues that interact with it^30,31^. Interestingly, such activating mutations have been identified as a cause for various cancers and RASopathies. In cancer, activating mutations in MEK itself have been proven to act as bona fide oncogenes in a limited number of cancers^32^. Initially only a small set of mutations were identified, including Q56P-MEK1 and K57N-MEK1 in lung cancer^33,34^, and D67N-MEK1 in ovarian cancer^35^. However, many other cancer-related mutations in the low-activity determining regulatory areas were recently detected in MEK1 as well as in MEK2^32,36^, likely inducing higher basal activity of these kinases. Several of these, as well as other MEK mutations have been found as contributors of resistance to Raf inhibitors in melanoma^37,38^. Interestingly, mutations in the low-activity conferring regulatory areas were found in RASopathies and AVM as well^10,39,40^. Some of these were similar to those seen in cancer, but some (nine mutations in RASopathy^41^ and two in AVM^10^) were specific to the developmental diseases.

The large number of similar activating mutations with distinct ability to induce different diseases raised the question of what might be the molecular mechanism that is responsible for the different mutation outcomes. Initially, it was thought that these differences are derived from the intrinsic activity of each of the mutants, and indeed results pointing at that direction were recently published^41^. However, some of the mutations do occur in more than one disease, and it was recently found that the correlation between the mutant’s activity and their effects on development is sometime disrupted^42^. Therefore, it became important to analyze the additional mechanisms by which these activating mutations confer their effects and induce the distinct pathologies. Here we chose several MEK1 and MEK2 mutations including (i) the MEK1-Q56P that is known to either induce lung cancer (32), AVM (9) or resistance to MEK-inhibitors treatment of melanoma (36) as well as its MEK2 equivalent MEK2-Q60P that plays a role in resistance to MEK-inhibitors (37). Importantly, these two mutations were not found in the CFC RASopathy^41^. For mutations that appear only in CFC but not in cancer we used the most prevalent CFC MEK mutation, MEK1-Y130C as well as its MEK2 equivalent Y134C^43–48^. These four mutations are representatives of two main mutation clusters of MEK^44^, residing on the N-lobe and the kinase domain of these two proteins. Although the two clusters are found in very distant sequence positions of the protein, they are structurally very close, keeping MEK in a tightly closed conformation that secures the very low basal activity of these proteins^44^. Using these mutants, we found that their intrinsic activity is indeed elevated, where the activity of the Q56/60 P mutants is higher than that of the Y130/134 C ones. These findings correlated well with extensive Molecular Dynamics (MD) simulations and free energy calculations, supporting the notion that at least in part, the effect is due to changes in the intrinsic activity of the mutants. Interestingly, the mutants had only a minor effect on basal ERK phosphorylation, and did not modulate any of the stimulated phosphorylation. We then studied the reason for this discrepancy and found that the two mutation types have additional abnormalities. Thus, the Q56/60 P mutations are further activated by stabilizing the MEK proteins, causing high expression and higher total activity of the mutants. In addition both types of mutants gain further activation by lower sensitivity to PP2A. Our results thus indicate that some MEK mutations exert their function not only due to their elevated intrinsic activity, but rather by modulation of regulatory elements such as dephosphorylation or protein stability.

## Results

### CFC and cancer-related mutations elevate MEK activity

**Figure 1:**
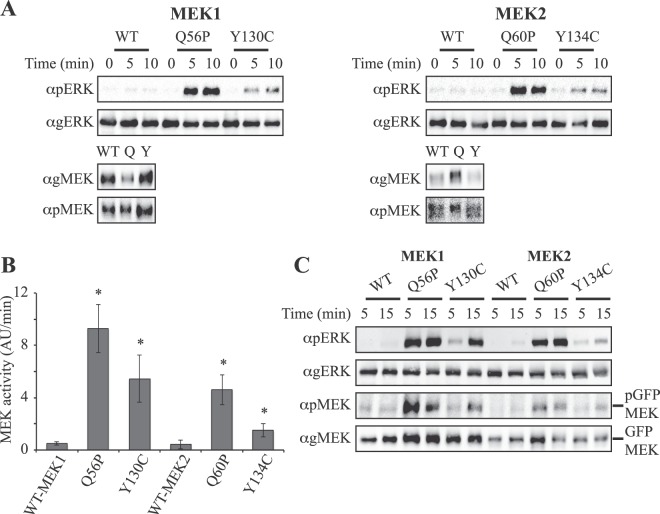
Effects of MEK1 and MEK2 mutations on their intrinsic kinase activity. (**A**) Intrinsic kinase activity of WT-MEK1/2 and their Q56/60 P and Y130/134 C mutants. Cos7 cells were transfected with the indicated GFP constructs, and 24 hours later the cells were serum starved for 14 hours after which the cells were harvested in Buffer H, sonicated (2 × 7 sec) and centrifuged, and the extracts were then subjected anion exchange mini-colons. Then the amount of protein was determined using anti pMEK and gMEK Abs, and the amount of phosphorylated MEK was equalized by adjusting the small amounts of phosphorylated MEK intensity (final amounts are shown in A bottom panels). This was done in order to follow changes in the intrinsic activity without the influence of the activatory phosphorylations. The partially purified MEKs were then used for *in-vitro* kinase assay using GST-ERK2 as a substrate (30 °C indicated times). The reaction was performed for 0, 5 and 10 min, after which the reaction was terminated with SB and boiling, and the sample were subjected to western blotting with anti-pERK and gERK Abs (A top panels). (**B**) Quantification of the results in (**A**). The catalytic activity of MEK was determined as the intensity of the ERK bands per min. The bar-graphs are averages and standard errors of 3 distinct experiments. *P < 0.01 according to student’s t-test. (**C**) *In vitro* ERK phosphorylation by IPed MEKs and their mutants. Cos7 cells were transfected with the indicated GFP constructs for 24 hours, serum starved for 14 hours and then the cells were harvested in Buffer H, sonicated (2 × 7 sec) and centrifuged. The MEKs and the mutants were IPed with anti GFP Ab, washed extensively, and then subjected to *in vitro* kinase assay using GST-ERK2 as a substrate (30 °C, indicated times). The reaction was terminated and the sample were subjected to western blotting with anti-pERK gERK, pMEK and gMEK Abs.

To investigate mechanisms by which distinct activating MEK mutations differentially induce CFC or cancer we used Q56P-MEK1 or Q60P-MEK2 that were mainly linked to cancer, and Y130C-MEK1 or Y134C-MEK2 that were found only in CFC. In order to confirm that the mutants are hyperactivated in cells, it was not sufficient to use bacterially expressed MEK1 or MEK2 that lack the post-translational modifications governing MEK activity. Therefore, we overexpressed WT-MEK1 and MEK2 as well as the four mutants in COS7 cells, followed by a partial purification of MEKs from non-stimulated cell lysates using DE-52 mini-columns^49^. The amount of phosphorylated MEK was equalized by adjusting the amounts of pMEK intensity (Fig. 1A bottom panels), in order to obtain information on changes in the intrinsic activity of the kinase, regardless of the minute activatory phosphorylations detected in resting cells. All mutations caused higher intrinsic MEK catalytic activity demonstrated by its ability to phosphorylate GST-ERK2 (Fig. 1A top panels), where that of Q56P-MEK1 and Q60P-MEK2 was higher than that of Y130C-MEK1 or Y134C-MEK2 mutants (Fig. 1B). Interestingly, the mutants of MEK1 were more active than those of MEK2. These results were corroborated by repeating the ERK phosphorylation with MEKs IPed from non-stimulated cells using Ab to the GFP at the C-terminus of overexpressed MEK (Fig. 1C). These results indicate that both mutation types significantly elevate the intrinsic activity of all four MEK mutations, where Q56P-MEK1 and Q60P-MEK2 activity is higher than that of Y130C-MEK1 or Y134C-MEK2.

### Molecular Dynamics (MD) and metadynamics simulations predict faster inactive-to-active transition in mutated MEKs

**Figure 2:**
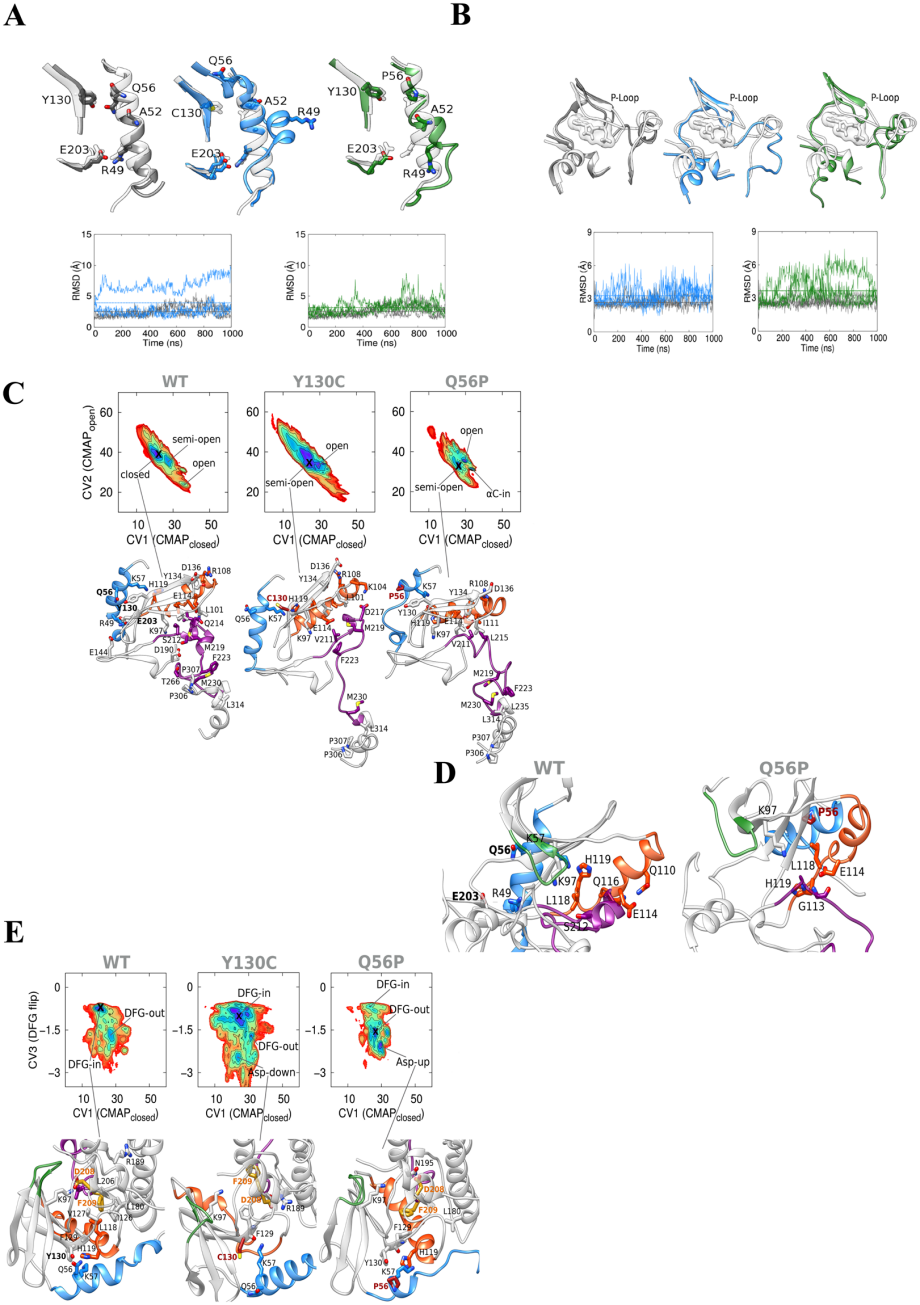
*In silico* simulations predict easier closed to open transition in mutated MEKs. (**A**) Dynamic effects of mutations on MEK1 αA-helix. (structures) αA-helix most representative structures for unphosphorylated apo WT, Y130C and Q56P MEK1 simulations (in gray, blue and green respectively). For Y130C mutant two representative structures (corresponding to the WT-like and odd simulations) are depicted. (graphs) RMSD of the αA-helix with respect to that in 3EQD MEK1 X-ray crystal structure: WT, Y130C, and Q56P (same color code as above). Dotted lines indicate the RMSD average value among the three simulations. (**B**) Effects of mutations on P-loop flexibility. (structures) P-loop most representative structures for unphosphorylated apo WT, Y130C and Q56P MEK1 simulations (same color code as above). (graphs) Residue RMSD of the P-loop with respect to 3EQD MEK1 X-ray crystal structure: WT, Y130C and Q56P. Dotted lines indicate the RMSD average value among the three simulations. (**C**) Free-energy surface of wild-type MEK1 and the three mutants as a function of CV1 (x-axis) and CV2 (y-axis). A cross indicates the global free energy minimum for which a representative structure is shown below. Each minimum is also named according to the corresponding main feature. The contour lines are drawn every 10 kJ/mol. In the structures below, the αA-helix is show in light blue, the αC-helix in orange, the A-loop in magenta. The mutated residues are shown in red. (**D**) Comparison of WT A-loop closed conformation with Q56P mutant structures of the free energy minima corresponding to the fully open state showing a partial αC-helix rotation toward the catalytic site (αC-in): the αA-helix is show in light blue, the P-loop in green, the αC-helix in orange, A-loop in magenta. The mutated residues are in red. (**E**) Free-energy surface of wild-type MEK1 and the three mutants as a function of CV1 (x-axis) and CV3 (y-axis). Each minimum is named according to the corresponding main feature. The contour lines are drawn every 10 kJ/mol. In the structures below, the αA-helix is show in light blue, the P-loop in green, the αC-helix in orange, the A-loop in magenta. DFG-motif D208 and F209 residues are shown in yellow, and the mutated residues in red.

In order to verify that the activity measured above is due to the intrinsic activity of MEK, and not caused by differential post-translational modifications, we turned to molecular dynamic simulations and free energy calculations. This approach was successfully used to predict the effect of a number of kinase mutations^50^ and should serve as a very reliable way to model the intrinsic MEK activity without influences of bacterial or cytosolic modification. In order to follow the inactive-to-active transition, 1-μs-long conventional MD simulations were performed on the unphosphorylated as well as phosphorylated ATP-bound state of WT-MEK1, as well as that of its Y130C and Q56P mutants. We found that both mutants favored the inactive-to-active transition in both the unphosphorylated apo and the phosphorylated ATP-bound MEK1 state. In the basal state, they increased αA-helix structural flexibility, promoting both its partial unfolding and the loose of αA-helix/core native contacts (e.g., Y130/Q56, R49/E203) (Figs 2A and S1A, Table 1). However, a distinct effect was found in Q56P with respect to Y130C mutant, involving the increase of P-loop flexibility, which could be causing a higher cofactor turnover (Fig. 2B). Over-activating effects were also found in the phosphorylated A-loop, where the two mutants induced an increase of the A-loop flexibility (Fig. S1B). Moreover, we observed a destabilization of both the αC-helix and the hydrogen bond between αC-helix E114 and A-loop Q214 residues, whose interacting frequency dropped from 85% in the WT to 29% and 54% for Y130C and Q56P mutant respectively (Fig. S1C, Table 1). This data suggests that both mutants could result in the acceleration of active-to-inactive process with significant impact on both the biological states analyzed.

**Table 1:** Average RMSD and contact frequency values along the MD simulations.

	RMSD_avg_ (Å)				Pair contact frequency (%)			
	αA-helix^a^	P-loop^a^	αC-helix^b^	A-loop^b^	A52/Q56^a^	Q56/Y130C^a^	R49/E203^a^	E114/Q214^b^
WT	2.5	2.6	3.8	4.7	84	76	80	83
Y130C	3.9	3.3	4.3	5.5	63	5.7	59	29
Q56P	3.2	3.6	4.3	5.7	0	18	62	54

A pair contact is defined as residue-residue minimal interatomic distance <4 Å; ^a^unphosphorylated apo MEK1 structure; ^b^phosphorylated ATP-bound MEK1 structure. RMSD values are referred to backbone atoms.

Next, four additional 1-μs-long simulations were run using PTmetaD-WTE protocol^50,51^, an enhanced sampling approach which combines parallel tempering with well-tempered metadynamics. In agreement with conventional MD, the free-energy landscapes obtained using PTMetaD-WTE confirmed that both mutations seemed to favor the inactive to active state transition, although to a different extent. Q56P-MEK1 showed significant effects on the closed-to-open transition of the A-loop, stabilizing an intermediate state in which the A-loop became completely unfolded. On the other hand, Y130C-MEK1 mutation showed milder effects (although clearly appreciable) on the stabilization of an intermediate state in which the A-loop is still partially folded (Fig. 2C). Regarding αC-helix flexibility, a higher propensity for out-to-in transition was observed only in the Q56P-MEK1 mutation (Fig. 2D). In addition, both mutants, although through different mechanisms, significantly flattened the energy barrier for the DFG motif in the MEK activation loop in-to-out transition, which could promote the ADP release and increase the ATP turnover rate (Fig. 2E). Together, the simulation results concur with the biochemical measurements of intrinsic activity. Both mutants were predicted to be more activated, Q56P-MEK1 to a higher extent than that predicted for Y130C-MEK1.

### The activating MEK mutations elevate the basal activity of ERK while stimulation increases MEK but not ERK phosphorylation

**Figure 3:**
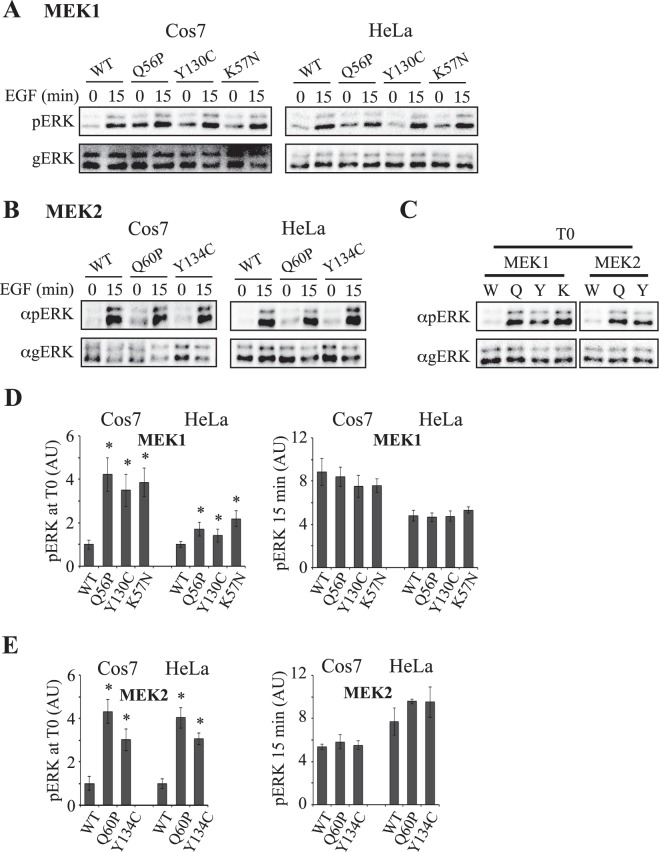
Effects of overexpression of MEKs and their mutants on ERK phosphorylation in Cos7 and HeLa cells. (**A**) Levels of ERK phosphorylation in cells transfected with MEK1 and its mutants. Cos7 or HeLa cells were transfected with GFP-MEK1 and its mutants, and 24 hours later the cells were serum starved (0.1% FBS, 18 h), and then stimulated with EGF (50 ng/ml) for 15 min or left untreated. Cytosolic extracts were prepared by sonications (2 × 7 sec.) and subjected to Western blotting with anti pERK and gERK Abs as indicated. (**B**) Levels of ERK phosphorylation in cells transfected with MEK2 and its mutants. Cos7 or HeLa cells were transfected with MEK2 and its mutants and then treated as described in (**A**). (**C**) Comparing the basal ERK phosphorylation of MEK1 and its mutants. The extract of the time 0 of each HeLa cell experiment in (**A**) and (**B**) were loaded side by side, and subjected to western blot with anti-pERK and gERK as indicated. (**D**,**E**) Quantification of the results in (**A**) and (**B**). The average intensity and standard errors of 3 experiments are displayed. *P < 0.05 according to student’s t-test.

We then undertook to examine the effect of the active mutants on ERK phosphorylation in cells. For this purpose, we transiently overexpressed WT-MEK1 and MEK2, the Q56P-MEK1, Y130C-MEK1, Q60P-MEK2 and Y134C-MEK2, as well as the cancer related K57N-MEK1 constructs, in COS7 or HeLa cells. Following pERK in the transfected cell lines, we found that basal ERK phosphorylation was increased by the 4 mutants (Fig. 3A–E), although the differences in pERK were much less pronounced than the differences in the intrinsic activity of the mutants. These effects were slightly stronger than the effect of the well-studied, intrinsically active, S218,222E mutant of MEK1^25^, while the inactive MEK1 (K97A^52^,) reduced this basal activity (Fig. S2). Moreover, in similarity to recent findings^53^, we found no significant variation in ERK phosphorylation upon stimulation between the mutants. Thus, EGF-stimulation elevated ERK phosphorylation, but in this case, there was no difference in the maximal effect between the WT protein and the activated mutants. This was similar to the results with S218,222E mutant of MEK1, while the K97A had inhibitory effect as expected (Fig. S2). This lack of change in maximal activity could have been due to either the ability of the activatory phosphorylation to overcome the small effects of the mutations tested, or due to other modes of regulation of the distinct mutants. Since the MD results indicated that the mutation can induce an additive effect to the phosphorylation, we assumed that the effects of the mutants on ERK activity is not only dependent on their intrinsic activity, but is probably regulated by additional factors.

**Figure 4:**
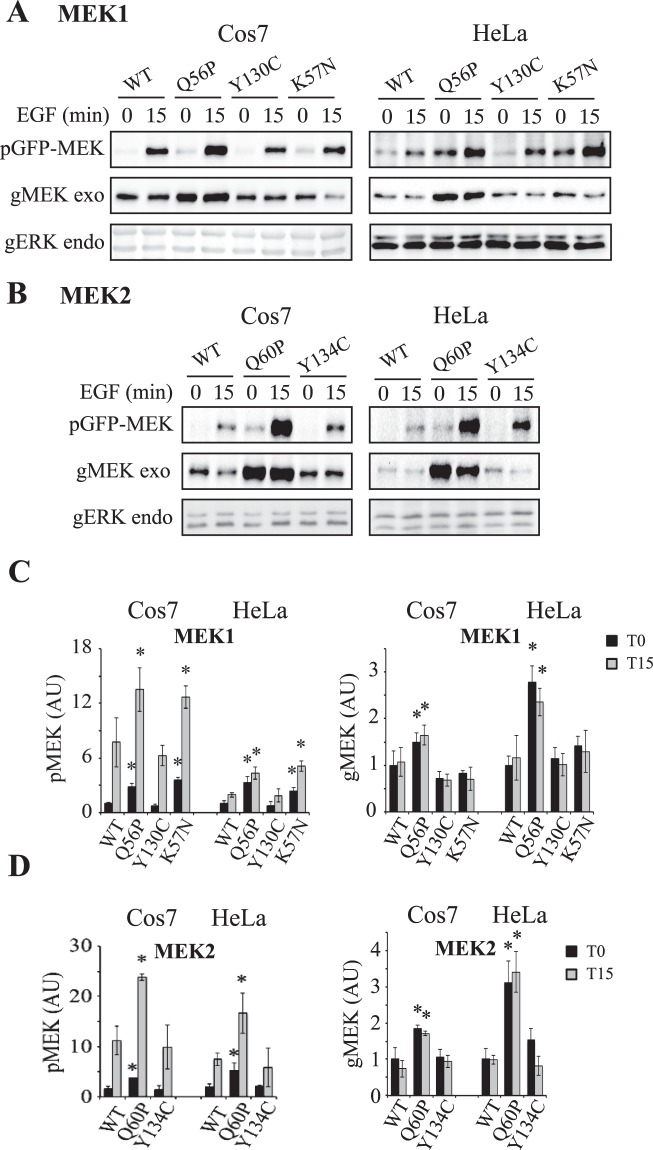
Effects of MEKs mutations on their phosphorylation and expression. (**A**) The phosphorylation and expression of MEK1 and its mutants. Cos7 and Hela cells were transfected with MEK1 and its mutants. The cells were then treated as described in Fig. 3 and blotted with anti pMEK, gMEK Abs and gERK Abs. The blotted endogenous gERK (bottom panels) is brought as a loading control. (**B**) The phosphorylation and expression of MEK2 and its mutants. Cos7 and Hela cells were transfected with MEK2 and its mutants. The cells were then treated as described in Fig. 3 and blotted with anti pMEK, gMEK and gERK Abs. The blotted endogenous gERK (bottom panels) is brought as a loading control. (**C and D**) Quantification of the results in **A** and **B**. The bar-graphs represent averages and standard errors of 3 experiments. **P < 0.05 according to student’s t-test.

In order to elucidate the other mechanisms and structural parameters that affect MEK activity by the distinct mutants, we first followed MEK expression and phosphorylation. Interestingly, we found that unlike ERK phosphorylation, the phosphorylation of MEK significantly differs between the various constructs. Thus, both basal and stimulated phosphorylation of MEK1 were significantly increased in the cancer–related mutations Q56P-MEK1 and K57N-MEK1, while there was no change or even a small decrease in the phosphorylation of Y130C-MEK1 as compared to WT control in both COS7 and HeLa cells (Fig. 4A,C). Similar results were obtained with the MEK2 mutants (Q60P and Y134C) as well, although in some experiments in HeLa cells, the phosphorylation of Y134C-MEK2 was higher than that of WT (Fig. 4B,D). Thus, no correlation was observed between the phosphorylation of the various MEKs and their activity or in-cell ERK phosphorylation. More surprising were the results with the amount of overexpressed MEK1 and MEK2, where we found that the expression of Q56P-MEK1 and Q60P-MEK2 was consistently higher than that of WT-MEK1/2 and the other mutants. These results indicate that although the total phosphorylation of Q56P-MEK1 and Q60P-MEK2 was increased, their specific phosphorylation was not significantly changed. In addition, the fact that the specific phosphorylation of K57N-MEK1 is increased (Fig. 4A,C), indicates that the two cancer-related mutations have increased phosphorylation achieved by distinct ways. Interestingly, the phosphorylation of HSF1 by MEKs^15^ did correlate to pMEK (Fig. S3), indicating that the regulation of this phosphorylation by MEK is distinct from that of ERK1/2.

### Characterization of MEK and ERK phosphorylations in cells transfected with MEK and its mutants

**Figure 5:**
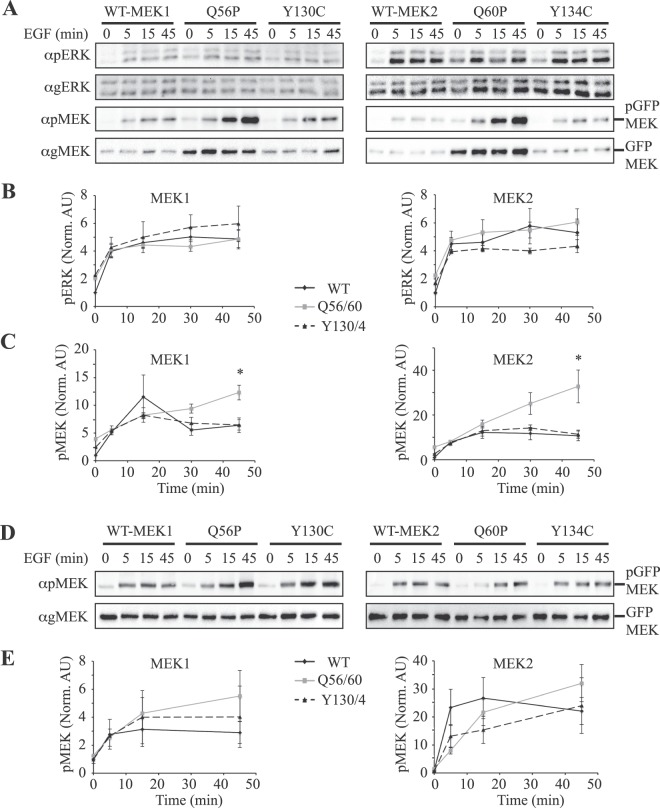
Effects of overexpression of MEKs and their mutants on ERK phosphorylation after longer EGF stimulation. (**A**) Effect of MEKs and their mutants on ERK and MEK phosphorylation and expression in Cos7 cells. Cos7 cells were transfected with the indicated plasmids. Twenty-four hours after transfection the cells were serum starved (0.1% FBS, 18 h), and then stimulated with EGF (50 ng/ml) for the indicated times, or left untreated. Cytosolic extracts (prepared by sonication 2 × 7 sec) were subjected to western blotting with anti pERK, gERK, pMEK, and gMEK Abs. (**B**,**C**) Quantification of the results in A for for pERK (**B**) or pMEK (**C**). The results are average and standard error of 3 experiments. *P < 0.05, according to student’s t-test. (**D**) Levels of phosphorylated MEKs when compared to equalized gMEK instead of equalized gERK. The extracts were equalized by gMEK in order to show the specific MEK phosphorylation. Western blots with anti pMEK and pMEK are presented. (**E**) Quantification of the results in D. The results presented are average and standard error of 3 experiments. *P < 0.05, according to student’s t-test.

We were interested to determine whether the differences in stimulated pERK occur at later time points therefore, we followed ERK and MEK activatory phosphorylations for up to 45 min after stimulation with EGF (Fig. 5A,B). Apparently, only the basal pERK levels were changed by the MEK mutants in the transfected cells (0), while no effect was seen at any time point tested after EGF stimulation (up to 45 min) in both COS7 and HeLa cells. Even later time points that were tested for up to 2 hours following EGF stimulation did not reveal any differences (data not shown). We did detect hyperphosphorylation of the activatory residues of the mutant MEK1 and MEK2, where those of the Q56/60 P mutation were more pronounced than that of Y130/134 C. Interestingly, the effects of the Q56/60P-MEKs were even more pronounced at 45 min after stimulation, when the expression of pQ56/60P-MEKs was 2–3 folds higher than that of WT-MEKs, while no changes with time were detected with the Y130/134 C mutants. As for the shorter time points above, we found that when the general loading were equalized according to gMEK (Fig. 5D,E), the level of pQ56/60 P MEKs was only slightly (but not significantly) higher than WT- or Y130/134C- MEKs. This indicates that the hyperphosphorylation, which is not affected even after lengthy stimulation, is not due to different regulation, but rather might be due to higher expression.

### MEK regulators play an activity-independent role in the mutant’s function

Although HSF1 phosphorylation correlated nicely with the activatory MEK phosphorylation, no such fit was found for ERK1/2 phosphorylation (Figs 3 and 5), indicating that this step is specifically affected by the mutations. To explain this lack of effect, we hypothesized that the excess activity of the mutants might be counteracted by an altered regulation of the MEK-ERK layer of the cascade. Since the binding site of MEK to ERK is localized in the very C-terminus of MEK^54^, it is unlikely that this effect is hindered by the mutations. To assess whether localization plays a role^55^, we examined the localization of the overexpressed MEKs, and found that both mutants localized similarly to WT-MEKs (Fig. S4). This was true both at resting cells and after EGF stimulation for MEK1, MEK2 and their mutants. Finally, we looked at the sensitivity to phosphatases, and found that at the relevant time scale upon EGF stimulation, there is no expression of MKP3, the main ERK phosphatase in the examined cells (Fig. S5). Thus, interaction, MEK localization and MKP3 are not involved in the effects of the MEK mutants, but we did find an influence of other regulators as follows:

### The high expression of Q56/60P mutants is due to a better stability

**Figure 6:**
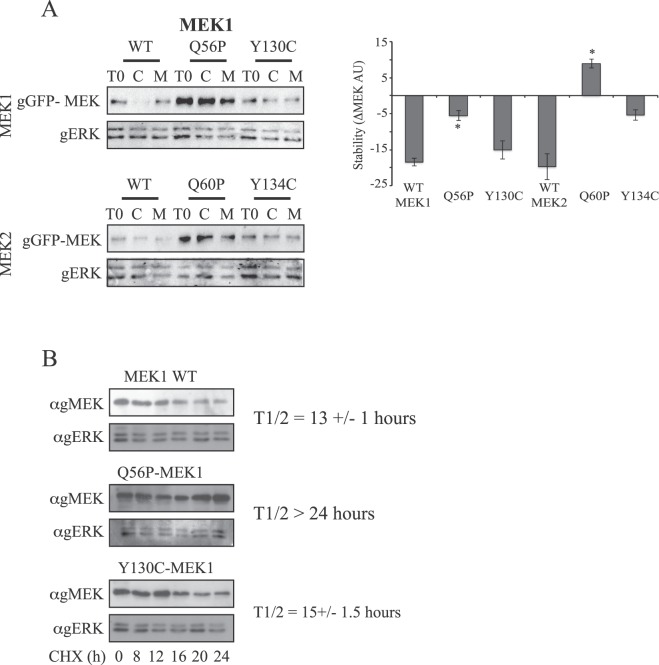
Stability and intrinsic activity of MEKs and their mutants. (**A**) Effect of translation and protease inhibitors on MEKs and their mutants. Cos7 cells were transfected with the indicated constructs, and 24 hours later the cells were treated with either Cyclohexamide (C; 1 μg/ml) or MG132 (M, 10 μM) for 8 h or left untreated. Then the cells were harvested with sonication (2 × 7 sec) and subjected to western blotting with anti gMEK and gERK as indicated. Quantification of the results appears in the lower panel. The bargraph represent the average and standard error of 2or 3 experiments *P < 0.05 by student’s t-test. (**B**) Effect of Cyclohexamide on the expression of WT-MEK1, Q56P-MEK1 and Y130C-MEK1. COS7 cells were transfected with WT-MEK1, Q56P MEK1 and Y130C-MEK1. Twenty-four hours later, the cells were treated with Cyclohexamide (CHX; 1 µg/ml) for the indicated times (bottom). The cells were then harvest and subjected to western blotting using anti gMEK and gERK Abs. The half-life of the GFP-MEK proteins is indicated in the right.

Since the expression of both Q56P-MEK1 and Q60P-MEK2 was higher than that of WT-MEKs, we examined whether this change is induced by higher protein stability of the mutants. For this purpose, we used cyclohexamide that prevents proteins translation and the proteasome inhibitor MG132. Thus, eight hours after addition of cyclohexamide to the cells, WT-MEK1/2 expression was significantly decreased, but no significant effect was detected with the cells treated with MG132 (Fig. 6A). Interestingly, the Q56/60 P MEKs were not affected at all by these inhibitors. When normalized to ERK amounts, the Q56P mutant of MEK1 is about three times more stable than the WT MEK1, and Q60P of MEK2 is degrading even less than the stable ERK1/2^56^. Time courses showed the proteolysis of WT-MEK1 and Y130C-MEK1 are gradual demonstrating expression half-life of 13+/−1 and 15+/− 1.5 hours respectively, while the Q56P-MEK1 mutant was much more stable and its expression half-life was more than 24 hours (Fig. 6B). Since the addition of the proteasome inhibitor MG132 did not increase MEK levels, it appears that MEK-GFP is degraded in a proteasome independent manner. High pMEK levels in Q56/60 P mutants, due to higher stability and expression, could explain the elevation in basal pERK observed in cells overexpressing these mutants. However, high basal pERK was also seen in cells overexpressing the Y130/134C mutants that do not show either general or phosphorylated MEK elevation, suggesting that additional mechanisms might regulate the WT-MEKs and their mutants.

### The MEK mutants are less sensitive to dephosphorylation by PP2A

**Figure 7:**
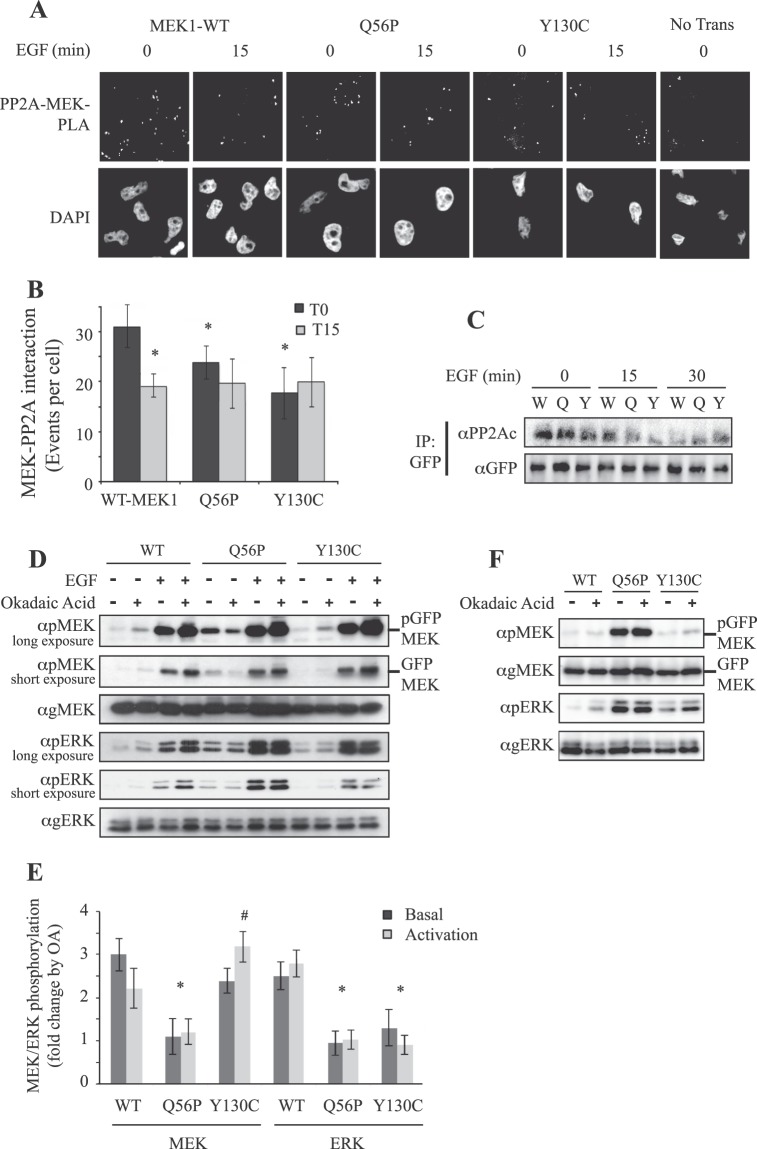
Effect of MEKs’ mutation on their interaction, as well as dephosphorylation by PP2A, and PP2A-induced effect on ERK phosphorylation. (**A**) Proximity ligation assay (PLA) examining the interaction between MEK-GFPs and PP2A. HeLa cells grown on coverslips were transfected with the relevant plasmids, and after 24 hours the cells were serum starved for 18 hours, and stimulated with EGF (50 ng/ml, 15 min) or left untreated. Cells were fixed, and subjected to a PLA assay according to the manufacturer’s instructions using the αGFP Ab together with the αPP2Ac Ab. Nuclei were detected with DAPI, and slides were visualized using a fluorescent microscope (X60 magnification). (**B**) Quantification of the results in **A**. Interaction-indicating fluorescent dots were manually counted within the GFP expressing population and presented as events per cell. The results are average and standard deviation of 30 distinct GFP-positive cells. Significance of *P < 0.05 according to student’s t-test was found for the differences between WT-MEK1 to stimulated WT-MEK1 or non-stimulated MEK mutants. No significant change was found between the stimulated WT-MEK1 compared to the stimulated interaction of the two mutants. (**C**) Interaction of MEK1 and its mutants with PP2A. Cos7 cells were transfected with the indicated MEK1 plasmids (W – WT, Q56P – Q, Y130C – Y), and 24 hours later cells were serum starved, and then stimulated with EGF (50 ng/ml) for 15 and 30 min. The cytosolic extracts were subjected to CoIP with anti GFP Ab, and the amount of interacting PP2Ac and the IPed constructs was determined by western blotting with anti PP2A and GFP Abs. (**D**) Effect of OA on MEK1 and its mutants in HeLa cells. HeLa cells were transfected with the indicated plasmids for 24 hours, followed by serum starvation (16 hours). The cells were then subjected to treatment with the PP2A inhibitor OA (1 μM, 20 min), or left untreated. Then, the indicated cell plates were stimulated with EGF (50 ng/ml, 15 min) and the rest were left untreated. The cytosolic extracts were subjected to Western blotting with the indicated Abs. (**E**) Quantification of the results in (**D**). Fold change of MEK and ERK phosphorylation in EGF-treated (activated) or non-treated (basal) between OA-treated to not-treated cells was determined. The average and standard error are from 3 experiments. *P < 0.05 of the indicated bars compared to WT. ^#^<0.05 of Y130C-MEK1 + OA compared to WT-MEK1 + OA. Both are calculated by student’s t-test. (**F**) Effect of okadaic acid on MEK1 and its mutants in Cos7 cells. Cos7 cells were transfected with the indicated plasmids for serum starved and treated with OA (1 μM for 20 min), or left untreated. The cytosolic extracts were subjected to Western blotting with the relevant Abs.

Unlike MKP3, we found that the catalytic subunit of the protein Ser/Thr phosphatase PP2A (PP2Ac) is expressed in all cells examined. Importantly, this protein showed differences in its interaction with stimulated MEK1 and its mutants (Fig. 7A,B). Thus, both Proximity Ligation Assay (PLA) and coimmunoprecipitation (Co-IP), demonstrated an interaction of PP2Ac with WT-MEK1, which was reduced after stimulation. The basal binding of Q56P-MEK1 and Y130C-MEK1 to PP2Ac was reduced, and this reduction was not changed 15 min after stimulation (Fig. 7A–C) but slightly increased 15 min later (Fig. 7C). The small reduction in interaction correlates with the elevated phosphorylation on the Q56/60 P MEKs in resting cells or elevated phosphorylation of Y130/134C-MEK1/2 in stimulated cells (Figs 4 and 5). Therefore, these results suggest a different mode of regulation of the MEKs and their mutants by PP2A. Moreover, the variable interactions and the fact that PP2A may dephosphorylate not only MEK but also ERK^57^ may explain, at least in part, why the differences observed in ERK1/2 phosphorylation at basal state do not appear after stimulation.

To confirm that PP2A is involved in the differential regulation of MEK and consequently ERK phosphorylations, we used the PP2A inhibitor, okadaic acid (OA). As expected, OA increased the activiatory phosphorylation of the overexpressed WT-MEK1 both before and after stimulation (Fig. 7D–F). This increase was reflected in ERK1/2 activation as well. The phosphorylation of Q56P-MEK1 was insensitive to OA treatment, neither under basal condition nor upon stimulation and this was reflected also at the ERK level. On the other hand, different effects were obtained with the Y130C-MEK1 that was activated by OA even stronger than WT-MEK1, while no OA-induced ERK activation was seen both before and after stimulation. Taken together, these results indicate that the reduced interaction of PP2A to Q56P-MEK1 results in its lack of sensitivity to OA, while that of Y130C-MEK1 does not affect the basal activity, but elevates MEK phosphorylation upon stimulation without changing ERK phosphorylation. Thus, MEKs, and their downstream ERKs differ in their sensitivity to phosphatases, which may result in a differential MEK and ERK downstream effects, under both basal and stimulated conditions.

## Discussion

Activating mutations of MEK1 and MEK2 are known to induce several human pathologies including cancer and developmental disorders^2^. Interestingly, the number of such mutations is higher than those found in most other signaling components, as not less than 20 MEK1 mutations and many other MEK2 ones were found in various human pathologies^41,46,47^ (and references therein). These mutations differ in the ability of their expressed proteins to activate ERK1/2 and to induce developmental or pathological phenotypes, but no direct correlation between the activity and the severity of the induced disease seems to exist^42^. Using two activating mutations of MEK1 and MEK2, we found that despite the pronounced differences in activity and phosphorylation between the WT and the mutated MEKs, there was only a relatively small difference in the basal activity of ERK, while no difference was detected upon stimulation. Here we investigated the other parameters that may influence the downstream effects of the mutants. Indeed, we found that the intrinsic activity is just one parameter that is changed, while other regulatory parameters such as stability or sensitivity to phosphatases affect the downstream activity as well. These might be the cellular contexts predicted to affect the emerging phenotypes in RASopathies of the distinct mutants^42^. For this study we used overexpression of WT MEK1/2 and their activating mutants in COS7 and HeLa cells. The expression of MEKs in both cell types was much higher than the expression of endogenous MEK (Fig. S2). The high level of MEK expression indicates that much of the ERK phosphorylation is derived from the mutated MEK, which mimics the situation in patients that express both WT and more so mutated MEK1.

It is generally difficult to obtain the information on the intrinsic activity of MEK free of post-translational modifications, as the expression of this protein, even in bacteria, results in its phosphorylation, which obviously changes the activity of the kinase. Our attempts to eliminate excess phosphorylation using phosphatases were not productive, as we were unable to remove all the incorporated phosphates. Therefore, we compared the activity of MEKs and their mutants after equalizing their minute basal phosphorylation. Significant elevation in the intrinsic activity of the mutants was observed using this method, but we could not rule out the possibility that some of these changes in activity might be due to additional phosphorylations or other post-translational modifications. In order to obtain another independent estimate of the changes in the intrinsic activity, we then resorted to MD simulations and free energy calculations. Using these methods, we observed that the changes in conformation caused by the mutations turn MEK more active, and the N terminal regulatory section less firm in preventing intrinsic activity. The results concurred with the *in vitro* activity measurements, as both mutants were predicted to be more activated, Q56P-MEK1 to a higher extent than Y130C- MEK1. These results nicely fall within what was expected from previous reports on these mutations^37,58^. All these data clearly indicate that Q56P-MEK1 has the highest intrinsic activity followed by Y130C-MEK1, while WT-MEK has very little intrinsic activity.

The consideration used for the MD experiments relied on the fact that, as virtually among all protein kinases, MEK1 active state conformation is characterized by a highly unpacked and extended conformation of A-loop, exhibiting a hairpin in its N-terminal region. Such peculiar orientation is fixed by a conserved hydrogen bond between the carboxyl of the D190 and the hydroxyl group of T226 (i.e., the aspartic acid of the HRD motif and the threonine of the conserved A-loop GT motif, respectively), which in turn is responsible for the correct orientation of the P-site hydroxyl acceptor group of the substrate during the MEK1/ERK phosphoryl transfer reaction^59^. Moreover αC-helix tilts toward the N-lobe, completing the active site and assuming the so-called *αC-in* conformation, which is sealed by a salt bridge between E114, the conserved glutamate of αC-helix, and K97, the catalytic lysine located on the β3-strand. Finally, the aspartate of the DFG motif assumes a conformation generally referred as *DFG-in*, facing its side chain into the ATP-binding pocket in order to coordinate the Mg2+ ion and properly orientate the ATP substrate. In the inactive conformation, this latter interaction is often disrupted by the turning of the DFG phenylalanine toward the ATP site (defined as DFG-out conformation) and is usually coupled with marked changes in A-loop, which adopts a highly packed and helical conformation, causing an extensive displacement of T226 hydroxyl group, which is placed about 9 Å away from D190 carboxyl. Finally, the αC-helix adopts the so-called *αC-out* conformation, rotating out of the catalytic cleft and tilts away from the N-terminal lobe. As a result, E114 side chain is unable to form the critical ion pair with the catalytic lysine, resulting in a hydrogen bond with the A-loop Q214 residue. In the extended molecular simulations, the Y130C mutant shows partially unfolded A-loop, in which residues D114 and K97 are closer (around 12 Å) than in WT (17 Å). In Q56P simulations, the most populated conformations show a much larger opening of A-loop than in Y130C, with E114 and K97 even closer (8.5 Å). Thus, the structural and energetic impact of Q56P is not only stronger than Y130C mutant, but also induces additional effects on the αC-helix displacement, not observed in Y130C simulations.

Although there were clear differences in intrinsic activity between the various constructs, in the context of the cell, the differences between pERK levels were detected only under basal conditions and not upon stimulation. As previously assumed^42^, these differences may have been the result of context-dependent regulation of MEKs, other than their intrinsic activity. Such regulatory components may include interaction with phosphatases, interaction with other kinases or scaffold proteins, distinct localization stability of MEK and ERK and distinct accessibility to substrates^60^. We examined all these possible regulators and our findings indicate that the activation of ERKs by the mutated MEKs is differentially regulated by enhanced protein stability of the Q56/60-MEKs, as well as increased interaction of the mutants with PP2A, but not the interaction with Raf (not shown) or negative feedback loops (e.g. Thr292 phosphorylation; (Fig. S2)). We also don’t think that the effect is due to limiting effects of ERK^61^, as overexpressed ERK gave similar results upon EGF stimulation to the endogenous pERK. However, we did rule out changes in localization, MEK-ERK interactions and MKP3. Thus, our results confirm that the MEK mutants are affected not only in their intrinsic activity but also by other regulatory processes.

The higher stability of the Q56 mutant may be explained by its resistance to proteasome-independent proteolysis, possibly in the lysosomes. However, not much is known regarding the mechanisms that regulate MEK stability, although it was previously shown that the scaffold protein p14 might attract MEK to the lysosome^62^. Although this attraction is to the lysosomal membrane, it is possible that some of the interacting MEK is degraded at that location. The resistance to lysosomal degradation is probably the cause of the mutant’s elevated expression, which together with the elevated intrinsic activity, results in higher levels of pMEK and elevated basal ERK activity. Another regulation that may affect the activation of MEK and ERK by the mutants is the interaction with PP2A. Here we show that the interaction of the MEK mutants with PP2A is lower than that of the WT-MEK (~30%), and that this mutant-PP2A interaction is not changed upon stimulation, bringing all of them to the same level. The reduced interaction causes an elevation of MEK1 phosphorylation in resting cells in Q56P-MEK1, but not in Y130C-MEK1. In addition, OA does not elevate the phosphorylation of Q56P-MEK1, while its effects on Y130C-MEK1 phosphorylation are either similar to WT in resting cells or elevated in stimulated ones (Fig. 7E).

The distinct effects between the two mutants may indicate that the regulation of their phosphorylation is mediated by different phosphatases. As not all molecules of PP2A are detached in the mutants, this can be due to distinct modes of interaction with, or regulation by, PP2A. For example, it is possible that the Q56P-MEK1 is losing a direct interaction with PP2A, while the Y130C-MEK1 loses the interaction with PP2A in upstream complexes such as KSR-induced one^63^. In addition, it is possible that the mutants have distinct sensitivity to free PP2A that does not bind to MEKs by docking interactions. More future studies are required to identify the exact mechanism in each case.

One of the questions that were raised by our results is the lack of effect of the mutants on the stimulated ERK phosphorylation. We showed that the effect of the mutants was seen only on the basal activity, which indicates that the pathological effects are induced by the basal and not stimulated activity. The differential interaction with PP2A can explain, at least in part this lack of elevated ERK activity in cells transfected with the mutants. The possibility that PP2A is brought by MEKs to the complex with ERK after stimulation indicates that PP2A may affect ERK phosphorylation as well. This may explain the fact that the stimulated phosphorylation of ERK by the mutant is not higher than that of the WT-MEK. Interestingly, unlike pERK, the phosphorylation of HSF1 did change after stimulation and correlated better with the activity of MEKs. This might be due to different regulation of this protein, which might be less susceptible to particular PP2As that interacted with MEKs.

In summary, in this study we characterized four activatory MEK mutants that serve as driver mutation in cancer (Q56/60P-MEK) or in CFC (Y130/134C-MEKs). We found that these mutants have higher basal activity than the WT-MEKs, and their downstream effects are also regulated by distinct stability and interaction with phosphatases. Thus, these results provide better understanding on the role of these mutants in disease such as cancer and CFC.

## Materials and Methods

### Cell culture, transfection and synchronization

COS7 and HeLa cells were cultured in Dulbecco’s modified Eagle’s medium (DMEM), supplemented with 2 mM L-glutamine, 1% penicillin-streptomycin and 10% fetal bovine serum (FBS), as previously described^64^. Cells were maintained at 37 °C in a humidified atmosphere of 95% air and 5% CO_2_. Cells were transfected with plasmids using polyethylenimine. Transfection efficiency was >80% in Cos7 cells and >70% in HeLa cells.

### Antibodies and reagents

Polyethylenimine, ATP, EGF, Cyclohexamide, and antibodies (Ab) against phosphorylated pERK, general ERK, general MEK and actin were purchased from Sigma-Aldrich. The antibody against pMEK was purchased from Cell signaling; Anti-GFP antibody mouse monoclonal antibody from Roche; rabbit polyclonal anti-GFP and anti p292-MEK1 Ab were from Abcam; Protein A/G PLUS–agarose beads and Abs against MKP3, and general HSF1 were obtained from Santa Cruz Biotechnology; pHSF1 was from Gene-Tex; and the antibody against PP2A (catalytic subunit) was from BD Transduction laboratories. DAPI was purchased from Invitrogen. The developing substrate NBT/BCIP was from Promega. Secondary Abs conjugated to horseradish peroxidase were from Jackson ImmunoResearch Laboratories; Okadaic acid and MG132 were purchased from Calbiochem. GST-ERK2 (in PGX vector) was prepared as previously described^65^.

### DNA constructs and mutations

*G*FP in pEGFP was purchased from Clontech. MEK1 was subcloned into pEGFP-N1 as described previously^49,66^ and MEK2 was subcloned into the same vector. Point mutations were performed by site-directed mutagenesis. GST–ERK2 were cloned in pGEX-2T vector (GE Healthcare, Buckinghamshire, UK) as previously described^67^, The GST protein was purified according to the manufacturer’s instructions and eluted from the glutathione beads using 10 mM of reduced glutathione.

### Cell harvesting and Co-IP

Transfected cells were grown to 70% confluence, and then serum-starved (0.1% FBS for 18 h). After stimulation or other treatments, cells were rinsed twice with ice-cold phosphate-buffered saline (PBS) and once with Buffer A (50 mM β-glycerophosphate pH 7.3, 1.5 mM EGTA, 1 mM EDTA, 1 mM dithiothreitol, and 0.1 mM sodium vanadate). The cells were then scraped into Buffer H (50 mM β-glycerophosphate pH 7.3, 1.5 mM EGTA, 1 mM EDTA, 1 mM dithiothreitol, 0.1 mM sodium vanadate, 10 μg/ml aprotinin, 10 μg/ml leupeptin, 2 μg/ml pepstatin A, and 1 mM Benzamidin) (0.4 ml/plate), sonicated (50 W, 2 × 7 s) and centrifuged (15,000 × *g*, 15 min). Supernatants were supplemented with sample buffer and boiled. For Co-IP supernatants were incubated for 2 hours (4 °C, with rotation) with protein-A/G–agarose beads (Santa Cruz Biotechnology) pre-linked with specific Abs (1 h, 23 °C). The bound protein-A/G beads were washed three times with ice-cold Buffer H with 30 mM NaCl (MEK with B-Raf and PP2A) or just buffer H. The IPed beads were resuspended in sample buffer and boiled; the resolved proteins were analyzed by western blotting with the indicated ABs.

### *In vitro* kinase assay

MEK was purified from transfected Cos7 cells by either Immunoprecipitation or anion exchange mini-columns. For Immunoprecipitation (IP), lysastes were added to protein-A/G–agarose beads pre-linked with Abs against GFP and incubated for 2 hours (4 °C, with rotation). The bound MEK-A/G beads were washed four times with Buffer A with 0.25 M NaCl and once with SAP buffer (50 mM Tris HCl pH 8.5, 5 mM MgSO_4_, 1 mM EGTA), resuspended in 120 μl SAP buffer and incubated with 2 units of SAP (Promega) for 30 min (30 °C, shaking). MEK-A/G beads were washed again, three times with Buffer A with 0.25 M NaCl and twice with Buffer A. Beads were then added into the phosphorylation reaction. For anion exchange, lysated were put twice through 400 μl dry volume of DE-52 beads pre-washed with 3 ml Buffer A. Flow through was collected and frozen (−80 °C) once a sample was taken for western blotting, to decide on amounts to use for the reaction equalizing pMEK. Once purified, MEK was incubated with GST-ERK2 (2 μg/reaction). Buffer RM (RM at threefold concentration is 30 mM MgCl_2_, 4.5 mM DTT, 75 mM β-glycerophosphate pH 7.3, 0.15 mM sodium vanadate, 3.75 mM EGTA, 30 μM calmidazolium, and 0.6 mM ATP), was added to the reaction at a final volume of 30 μl per time point and incubated at 30 °C with shaking. Samples were collected at the indicated times, and the reaction was terminated by adding 10 μl of 4x sample buffer, and boiling. Proteins were resolved and subjected to western blot analysis with anti-pERK and gERK Abs.

### Fluorescence microscopy

MEK-GFP transfected cells were stimulated for indicated times, or left untreated, then fixed in 3% paraformaldehyde in PBS (20 min, 23 °C), and incubated with 2% bovine serum albumin (BSA) in PBS (15 min, 23 °C), followed by permeabilization with Triton X-100 (0.1% in PBS, 5 min, 23 °C). The fixed cells were then washed three times with PBS and incubated with DAPI^68^. Slides were visualized by using spinning disc confocal microscopy (x60 magnification Zeiss, Jena, Germany). Background correction and contrast adjustment of raw data images were performed, using Photoshop (Adobe, CA).

### Proximity ligation assay

Protein–protein interactions were detected by using a Duolink PLA kit [Olink Bioscience, Uppsala, Sweden^69^;), according to the manufacturer’s protocol. Briefly, cells were grown, fixed, and permeabilized, as described in the immunofluorescence microscopy section. The samples were then incubated with primary Abs against PP2Ac and GFP (1 hour, 23 °C), washed (0.01 M Tris-HCl, pH 7.4, 0.15 M NaCl, and 0.05% Tween 20), and then incubated with specific probes (1 h, 37 °C), followed by DAPI staining to visualize nuclei and then another wash (0.2 M Tris-HCl pH 7.5, 0.15 M NaCl). The signal was visualized as distinct fluorescent spots by using spinning disc confocal microscopy. Positive cells were counted manually and their percent out of GFP positive cells was calculated.

### Statistical analysis

Data are expressed as mean ± s.e.m. Statistical evaluation was carried out using functional analysis and student’s *t*-test (two-tailed) to test for differences between the control and experimental results. Values of *P* < 0.05 were considered statistically significant.

### Structural modeling of MEK1 mutants

All the MEK1 variants studied here (i.e., WT, Y130C and Q56P; either in the unphosphorylated apo or in the phosphorylated ATP-bound form) were modeled based on the crystal structure of MEK1 in an inactive conformation (see Supplementary Information). The active conformation of MEK1 was modeled by homology based on the human MST3 kinase X-ray structure, which shows the highest sequence similarity to MEK1 among the STE kinases for which a canonical active structure is available in the PDB.

### Standard Molecular Dynamics (MD)

Three independent 1-μs MD simulations were run for each MEK1 variant either in the unphosphorylated apo or phosphorylated ATP-bound state, using GROMACS 4.6.7 program^70^. Overall, a total of 24 1-μs-long unbiased trajectories were collected. For each system, the three independent trajectories were merged and clustered, in order to select the representative structures.

### Metadynamics simulations

Parallel tempering metadynamics simulations combined with Well-Tempered Ensemble (PTMetaD-WTE) were performed exclusively on the unphosphorylated apo states. Two collective variables (CV1 and CV2) were used to study the inactive-to-active transition of the A-loop, namely the distance in contact map space to the closed and open A-loop conformation (as defined before^50^). As the DFG motif was found in an in-conformation in both the inactive and active reference structures used herein and given its relevance as hallmark to define kinases activity states, an additional collective variable (CV3) was specified describing its in-to-out transition. The free energy surfaces (FES) of the WT and the three mutants were obtained for the replica at 300 K by integrating the deposited bias during the PTMetaD-WTE simulations, as required by the metadynamics algorithm. For convenience, they are shown as function of two CVs at a time (CV1, CV2 and CV1, CV3). More details in Supplementary Information.

## Electronic supplementary material

Supplementary figures
