# Supplementary material for: Intrinsically active MEK variants are differentially regulated by proteinases and phosphatases
Source: Sci Rep. 2018 Aug 7;8:11830. doi: 10.1038/s41598-018-30202-5 (PMC6081382; doi:10.1038/s41598-018-30202-5)
Supplement: Supplementary file 1 — Supplementary figures [file 41598_2018_30202_MOESM1_ESM.pdf]

Intrinsically active MEK variants are differentially regulated by proteinases and phosphatases.

Merav Ordan, Chiara Pallara, Galia Maik-Rachline, Tamar Hanoch, Francesco Luigi Gervasio, Fabian Glaser, Juan Fernandez-Recio and Rony Seger

Supplementary figures

A

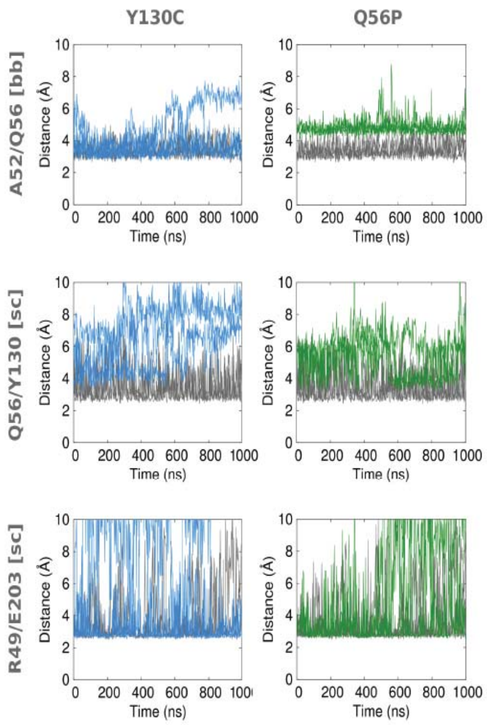
Y130C
Q56P
A52/Q56 [bb]
Q56/Y130 [sc]
R49/E203 [sc]
Distance (Å)
Time (ns)

B

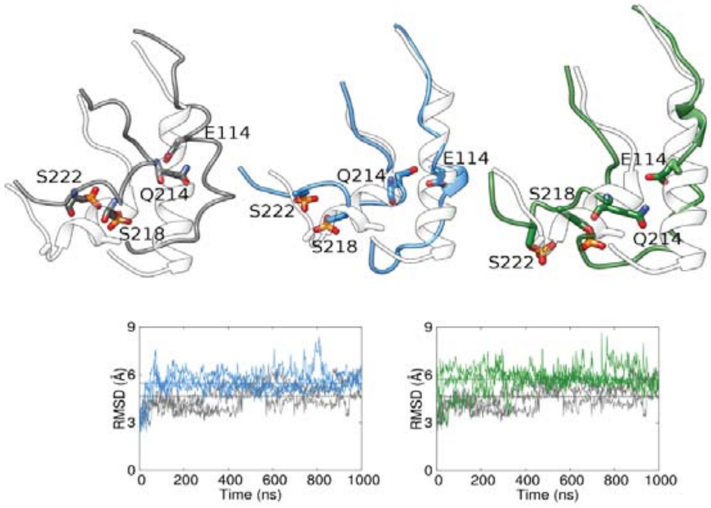
E114
S222
Q214
S218
RMSD (Å)
Time (ns)

C

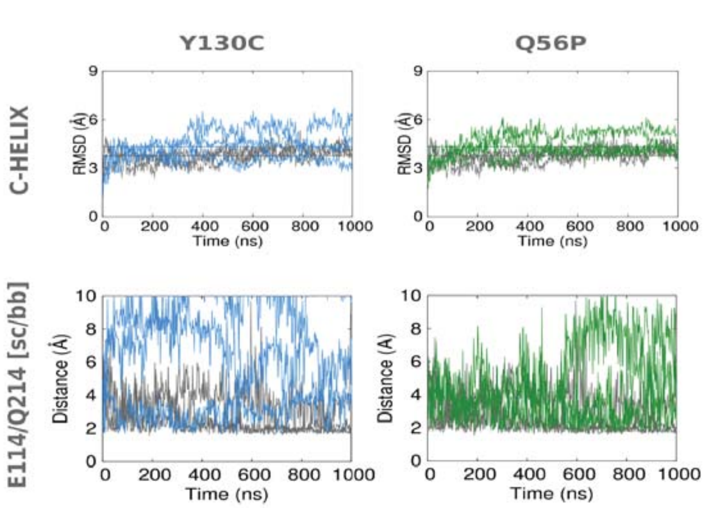
Y130C
Q56P
C-HELIX
E114/Q214 [sc/bb]
RMSD (Å)
Distance (Å)
Time (ns)

Figure S1. Molecular dynamics simulations. (A) Molecular interaction between αA-helix and kinase core. Residue-residue minimal distance of A52/Q56, Q56/Y130C and R49/E203 contacts. WT, Y130C and Q56P unphosphorylated apo MEK1 data are represented in gray, blue and green respectively. **(B)** Effects of mutations on A-loop flexibility. (structures) A-loop most representative structures for unphosphorylated apo WT, Y130C and Q56P MEK1 simulations (same color code as above). (graphs) RMSD of the A-loop with respect to that in 3EQD MEK1 X-ray crystal structure for WT, Y130C an Q56P (same color code as above). Dotted lines indicate the RMSD average value among the three simulations. **(C)** Effects of mutations on αC-helix flexibility. Top - αC-helix RMSD with respect to the X-ray crystal structure (3EQD) of MEK1 inactive state; Bottom - Minimal distance between Q214 backbone and E114 side chain residues. WT, Y130C and Q56P phosphorylated ATP-bound MEK1 data are in gray, blue and green respectively.

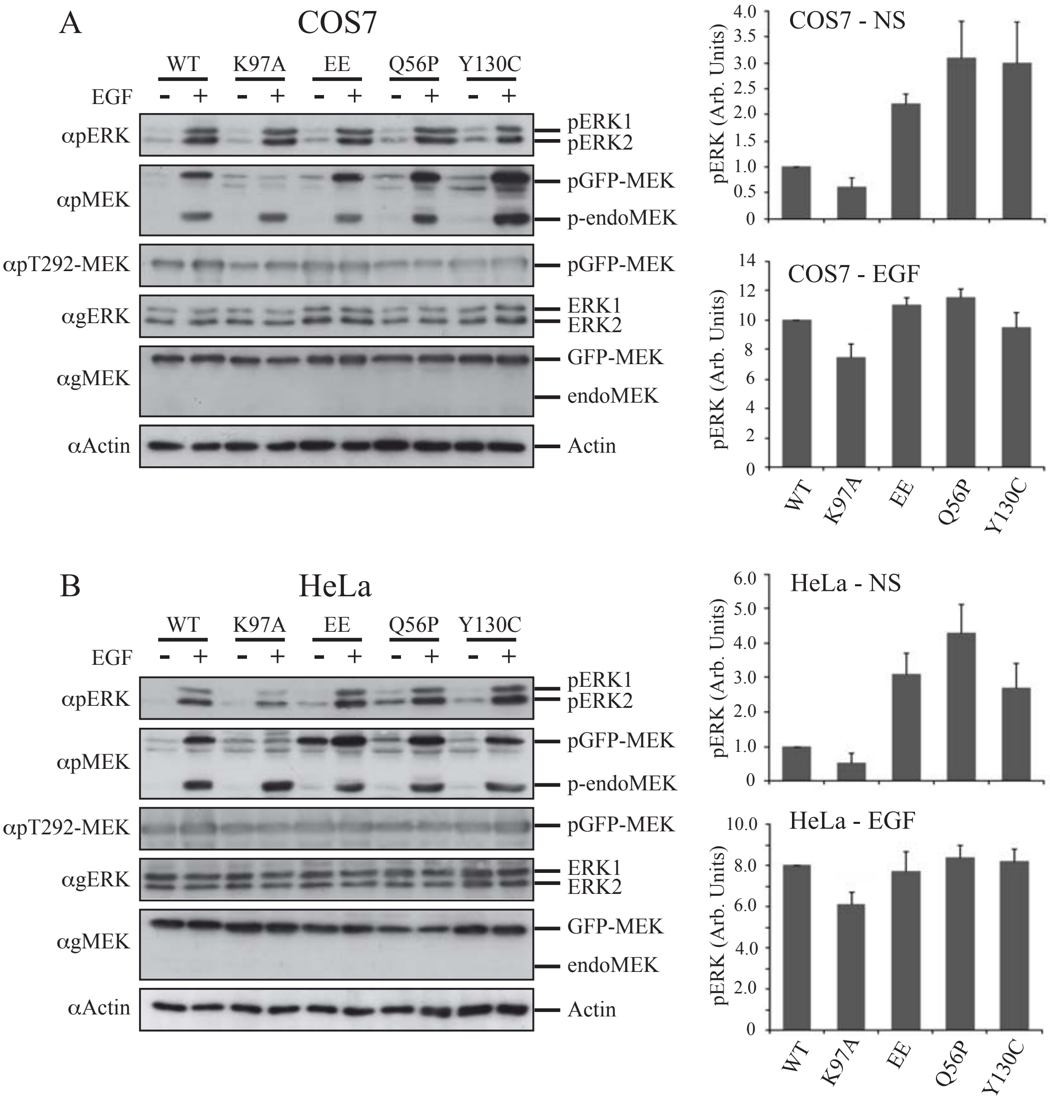

Figure S2. Comparison of the effects of WT MEK1 and the CFC and cancer-related mutants of MEK1 with K97A-MEK1 and S218,222E-MEK1. Cos7 **(A) and** HeLa **(B)** cells were transfected with the indicated plasmids for 24 hours, followed by serum starvation (16 hours). Then the cells were either stimulated with EGF (50 ng/ml, 15 min (+)) or left untreated (-). Cytosolic extracts (prepared by sonication 2x7 sec) were subjected to western blotting with anti-pERK, anti pMEK (S222,218) pT292-MEK, gERK, pMEK, and gMEK Abs. The average intensity and standard errors of 2 or 3 experiments for each condition is shown in the right side of the S2A and S2B.

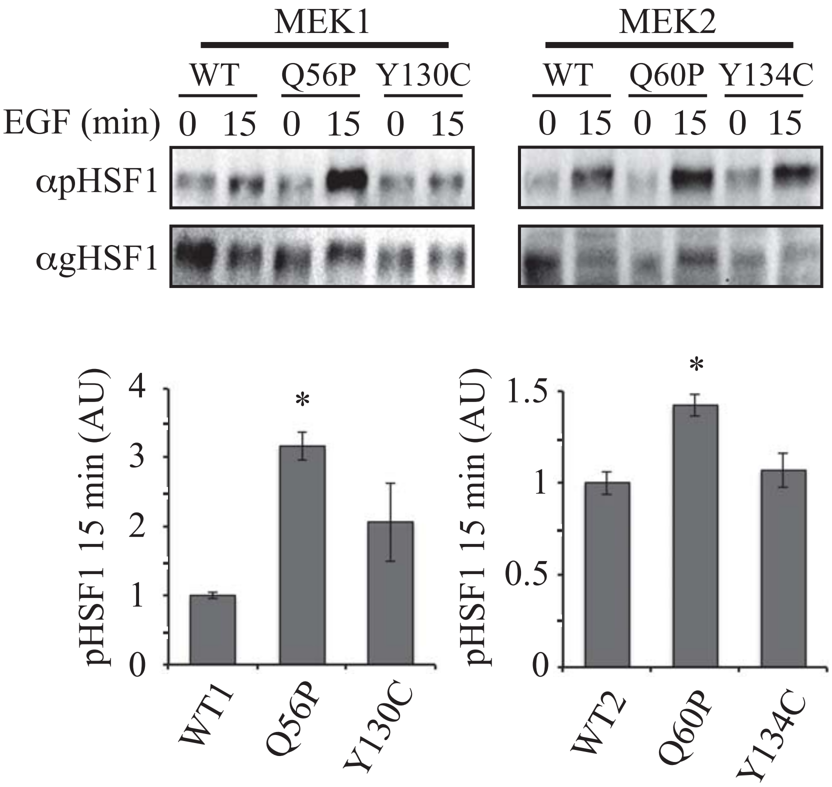

Figure S3. Effect of MEK1 mutations on pHSF1 phosphorylation. Cos7 cells were transfected with the relevant plasmids, and 24 hours later the cells were serum starved and then stimulated with EGF (50 ng/ml) for 15 min, or left untreated. The cytosolic extracts were subjected to western blotting with αpHSF1 as well as αgHSF1 Abs. Levels of pHSF1 at 15 min after stimulation were quantified, and the bar-graphs represent averages and standard error of 2 experiments. $P<0.05$ according to students' T test.

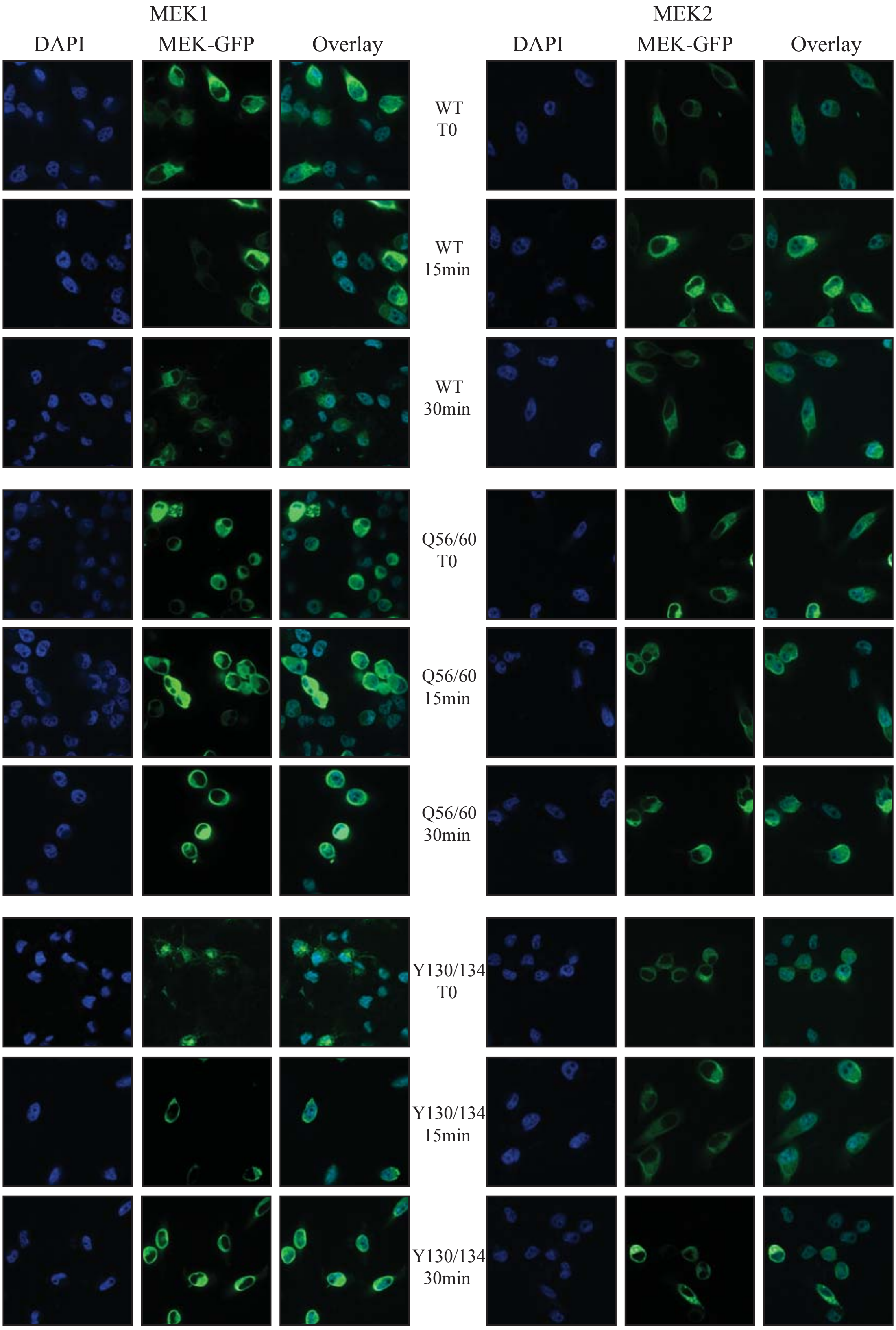

Figure S4. Localization of MEK-GFP is the same for WT and mutants. HeLa cells grown on coverslips were transfected with the relevant MEK-GFP plasmids, and 24 hours later the cells were serum starved, stimulated with EGF (50 ng/ml) for the indicated times, or left untreated, and then fixed. Nuclei were detected with DAPI, and slides were visualized using a fluorescent microscope (X60 magnification).

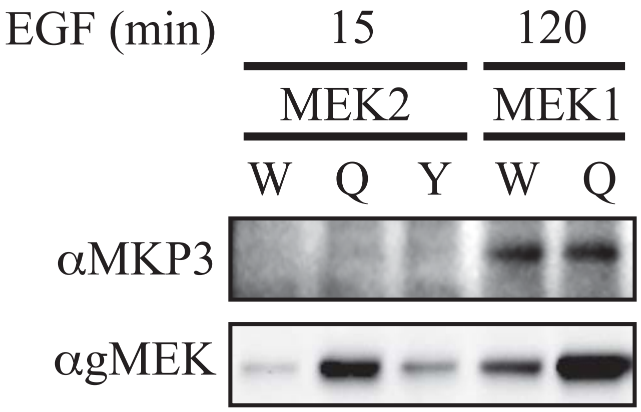

Figure S5. No expression of MKP3 in Cos7 cells at relevant times. Cells were transfected with the relevant plasmids, and 24 hours the cells were serum starved, and then stimulated with EGF (50 ng/ml) for the indicated times. Cytosolic extracts were subjected to Western blotting with αMKP3 as well as αgMEK Abs.
